# Supplementary material for: A comparison of the effects of two protocols of concurrent resistance and aerobic training on physical fitness in middle school students
Source: PeerJ. 2024 Apr 23;12:e17294. doi: 10.7717/peerj.17294 (PMC11048074; doi:10.7717/peerj.17294)
Supplement: Supplemental Information 2 [file peerj-12-17294-s002.doc]

**Supplementary Table 1. Details of the combined physical training prescription.**

|  | CT-0h group | CT-48h group |
| --- | --- | --- |
| Aerobic training |  |  |
| Type | Walking/running on the track | Walking/running on the track |
| Frequency | 2 days per week | 1 days per week |
| Warm up | 3 ~5 min with 50% V_max_ | 3~5 min with 50% V_max_ |
| Intensity | >75~85% HRmax interspersed with 50% V_max_ | >75~85% HRmax interspersed with 50% V_max_ |
| Progression | 5 x 1:1 min | 2 x 5 x 1:1 min |
| Back to calm | 5 min with 50% V_max_ | 5 min with 50% V_max_ |
|  |  |  |
| Resistance training |  |  |
| Tipo | 8 exercises involving the main muscle groups (articulated bench press, rowing sitting, triceps pulley, biceps curl, leg press 45°, horizontal leg, seated flexor, and sit-ups) | 8 exercises involving the main muscle groups (articulated bench press, rowing sitting, triceps pulley, biceps curl, leg press 45°, horizontal leg, seated flexor, and sit-ups) |
| Frequency | 2 days per week | 1 days per week |
| Time / Progression | Weeks 1-4: 15-20 RM  Weeks 5-8: 10-12 RM  Weeks 9-12: 8-10 RM | Weeks 1-4: 15-20 RM  Weeks 5-8: 10-12 RM  Weeks 9-12: 8-10 RM |
| Sets | 1 | 2 |
| Interval between sets and exercises | 60s | 60s |
| Order of exercises | Upper limbs before lower limbs | Upper limbs before lower limbs |
| Cool down | 5 min stretching involving the major muscle groups | 5 min stretching involving the major muscle groups |
| Total duration of combined session | 35~40 min | 35 ~ 40 min |
